# Tunable Chiral Terahertz Wave Absorption and Beam Manipulation Based on Vanadium Dioxide Metasurfaces

**DOI:** 10.3390/nano16030189

**Published:** 2026-01-30

**Authors:** Li Luo, Boyu Chen, Jie Li, Yi Zheng, Jin He, Yuanyuan Lv, Lin Liu, Cheng Chen, Jialuo Ding, Xiang Yan, Junqi Chen, Tian Tian, Zhe Zhao, Zhanyi Lin, Menghan Chen, Lin Liang, Jianquan Yao

**Affiliations:** 1 Sichuan Province Key Laboratory of Optoelectronic Sensor Devices and Systems, College of Optoelectronic Engineering (Chengdu IC Valley Industrial College), Chengdu University of Information Technology, Chengdu 610225, China; rolly2002_ll@163.com (L.L.); chenboyu0405@163.com (B.C.); cuithejin@163.com (J.H.);; 2 Sichuan Meteorological Optoelectronic Sensor Technology and Application Engineering Research Center, Chengdu University of Information Technology, Chengdu 610225, China; 3 Yunnan Provincial Weather Modification Center, Kunming 650034, China; zyaaav@163.com; 4 Information Center of Sichuan Provincial Commerce Department, Chengdu 610081, China; 5 School of Precision Instruments and Opto-Electronics Engineering, Tianjin University, Tianjin 300072, China

**Keywords:** tunable metasurface, circular dichroism, vanadium dioxide, beam manipulation

## Abstract

Chiral metasurfaces exhibit enormous potential in optical applications, and their integration with phase-change material vanadium dioxide (VO_2_) provides a novel pathway for dynamic regulation. In this study, a chiral absorptive metasurface based on VO_2_ is designed. By tuning the VO_2_ conductivity around the operating frequency of 2.81 THz, the circular dichroism (CD) can be continuously adjusted from 0.06 to 0.95, realizing a high-contrast chiral switch. On this basis, the Pancharatnam–Berry (PB) phase is introduced to construct a chirality-dependent phase gradient: when the VO_2_ conductivity is 200,000 S/m, only the left-handed circularly polarized (LCP) wave is subjected to periodic phase modulation, enabling controllable deflection of the reflected beam, while the right-handed circularly polarized (RCP) wave is selectively absorbed. This “chiral phase encoding” strategy simultaneously achieves absorptive CD tuning and reflective beam shaping on a single metasurface, significantly enhancing the flexible manipulation capability of circular polarization states in the terahertz band. It provides a compact and efficient solution for reconfigurable imaging, unidirectional communication, and integrated photonics systems.

## 1. Introduction

As a two-dimensional planar structure of metamaterials, metasurfaces are artificially designed subwavelength micro/nanostructure arrays with periodic or quasi-periodic arrangements, exhibiting unique and powerful capabilities in regulating electromagnetic waves [1,2,3,4]. Chirality refers to the characteristic that a unit structure cannot coincide with its mirror image after translation or rotation. Due to the chiral nature of the structure, its interaction with LCP and RCP waves exhibits chirality-dependent differential responses: specifically, differences in amplitude and phase responses to LCP and RCP waves [5,6]. These differences can be normalized and characterized by parameters such as CD [7,8,9] and optical activity [10,11,12]. CD is widely used in biomedical [13], chemical [14], terahertz communication [15], and other fields.

For traditional metal-based terahertz chiral absorptive metasurfaces, the CD performance is fixed once the structure is determined, which limits their practical application scenarios to a certain extent. Therefore, vanadium dioxide (VO_2_), a phase-change material, has been introduced into metasurface structures in the field [16,17,18,19,20]: VO_2_ is a temperature-responsive phase-change material that undergoes a Metal–Insulator Transition (MIT) around room temperature (approximately 68 °C). This phase transition causes significant mutations in its optical, electrical, and thermal properties. Utilizing this characteristic, the transition of VO_2_ between the insulating phase and the metallic phase is regulated by temperature or voltage excitation, thereby realizing the dynamic tunability of the chiral absorption performance of the metasurface. The Metal–Insulator–Metal (MIM) type metasurface has unique advantages in regulating the amplitude and phase of electromagnetic waves, making it an ideal structural system for constructing tunable metasurfaces. In recent years, VO_2_-based dynamically tunable terahertz metasurfaces have been extensively studied, showing promising application prospects in absorbers, polarization converters, and multifunctional integrated devices. In 2023, Xu et al. [21] proposed a single-layer graphene-based tunable metasurface, achieving a linear dichroism (LD) of 0.9 and a CD of 0.8 by using the incident angle of electromagnetic waves instead of structural chirality, and realizing bifunctional wide-range tuning by regulating the Fermi level of graphene; in the same year, Zhao et al. [22] proposed a VO_2_-based MIM structured tunable metasurface, which achieves the photonic spin Hall effect by combining the geometric phase in the insulating state, and realizes complete absorption of LCP waves and deflection of RCP waves in the metallic state, with a simple and easy-to-prepare structure; in 2025, Zhuang et al. [23] designed a VO_2_-based multifunctional terahertz metasurface. In the metallic state, it exhibited dual-band absorption-type linear dichroism (LD_A) and broadband absorption-type circular dichroism (CD_A); in the insulating state, it switched to multi-band transmission-type LD_T and dual-band transmission-type CD_T, supporting dynamic switching between absorption and transmission modes; in the same year, Zheng et al. [24] reported an S-shaped chiral metasurface absorber on a VO_2_ substrate, achieving dual-band CD effects (peaks of 0.814 and 0.784) at 0.871 THz and 1.365 THz, and obtaining high modulation depths (0.807 and 0.776) by regulating the VO_2_ conductivity; however, most of these studies have limitations such as scattered core functions, insufficient single chiral regulation efficiency, or regulation parameters relying on additional parameters.

To address these issues, this study proposes a chiral metasurface structure with an MIM configuration, incorporating the functionality of the phase-change material vanadium dioxide (VO_2_) into the metasurface. Relying on the Metal–Insulator Transition (MIT) characteristic of VO_2_, it breaks through the fixed limitation of CD performance of traditional chiral metasurfaces, realizing a dynamically tunable chiral absorptive metasurface with better single-frequency chirality, deeper modulation depth, more focused and practical function integration, and a relatively simple structure: the chiral absorption response of the structure is directly regulated by the phase state of VO_2_. When VO_2_ is in the “off” state, the CD effect of the metasurface at a specific operating frequency is weak; when VO_2_ transitions to the “on” state (metallic phase), the CD effect at this frequency is significantly enhanced with a large modulation amplitude, achieving a rare high-amplitude chiral absorption tunability in the terahertz band. Based on the Pancharatnam–Berry (PB) phase regulation principle [25,26,27,28,29], a chirality-dependent phase gradient metasurface is designed, realizing the dynamic switching of the metasurface from full chiral wave regulation to chiral selective regulation during the state transition of VO_2_ from “off” (insulating state) to “on” (metallic state).

## 2. Structural Design and Theoretical Analysis

### 2.1. Structural Design

**Figure 1:**
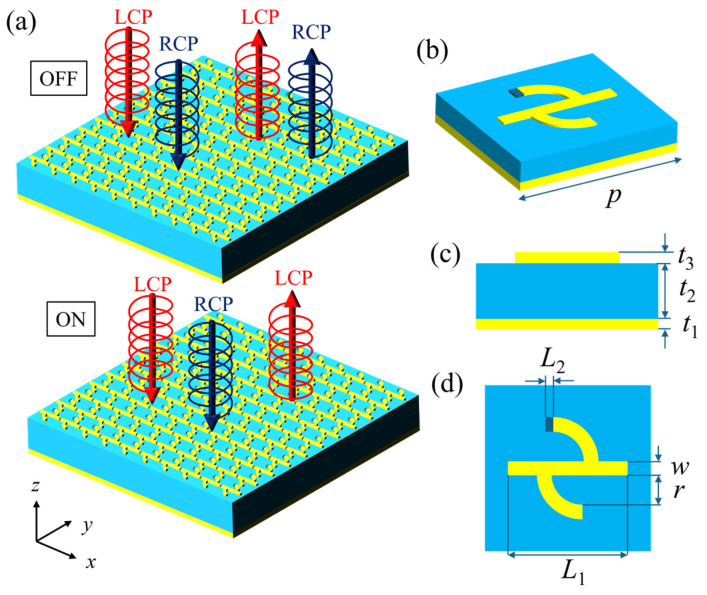
Schematic diagram of the tunable chiral metasurface structure. (**a**) Three-dimensional array functional schematic of the metasurface; (**b**–**d**) unit structure schematic diagrams.

The designed chiral metasurface is shown in Figure 1, which consists of three stacked layers: a top pattern arranged in a periodic array, a dielectric layer, and a gold substrate. The top structure is a periodically arranged chiral pattern with a thickness *t*_3_ = 1 μm and a width *w* = 4 μm, composed of 1 rectangular metal strip, 2 quarter-circular arcs (yellow region) and 1 rectangular VO_2_ patch (dark blue region). The parameters are *L*_1_ = 35 μm, *r* = 14 μm, *L*_2_ = 3 μm. Among them, the VO_2_ patch is the core functional structure for realizing phase-change regulation. The middle dielectric layer is a polyimide (PI) spacer layer (blue region) [30] (dielectric constant: *ε* = 3.5, loss tangent: tan *δ* = 0.0027) with a thickness *t*_2_ = 14 μm. Polyimide is a high-performance polymer with excellent properties, whose function is to add a buffer and heat insulation layer between the top and bottom layers. To suppress the transmission of electromagnetic waves, the thickness of the bottom reflective gold film is set to *t*_1_ = 0.1 μm, and the period of the entire supercell is *P* = 50 μm. Note that all materials used except VO_2_ are taken from the material library built into the electromagnetic simulation software, and the conductivity of VO_2_ is set according to its phase transition.

### 2.2. Theoretical Analysis

VO_2_ is used as the phase-change material, which can undergo a metal–insulator transition at a temperature of 68 °C. This transition of VO_2_ leads to a sharp change in conductivity, with changes of 3 to 4 orders of magnitude. When VO_2_ is in the insulating state, the conductivity *σ* = 2 × 10^2^ S/m, and when VO_2_ is in the metallic state, the conductivity *σ* = 2 × 10^5^ S/m. In the terahertz (THz) frequency range, the dielectric constant is usually described by the Drude model [31,32]:

(1) $$\varepsilon (\omega )=\varepsilon^{\prime }+\varepsilon^{\prime \prime }=\varepsilon_{\infty }-\left[\frac{\sigma \omega_{p}^{2}\left(\sigma_{0}\right)}{\sigma_{0}(\omega^{2}+i\gamma \omega )}\right]$$

In the formula, *ε*_∞_ = 12 is the high-frequency dielectric constant, *γ* = 5.75 × 10^13^ rad/s is the electron collision rate, *σ* is the VO_2_ conductivity, *σ*_0_ = 3 × 10^5^ S/m, the plasma frequency *ω_p_*(*σ*_0_) = 1.4 × 10^15^ rad/s, and *ω_p_*(*σ*) and *σ* are proportional to the free carrier density. The relationship between the plasma frequency and conductivity can be expressed as follows:

(2) $$\omega_{p}^{2}\left(\sigma \right)=\frac{\sigma }{\sigma_{0}}\omega_{p}^{2}\left(\sigma_{0}\right)$$

In the formula, the reference conductivity is set to *σ*_0_ = 3 × 10^5^ S/m, and the reference plasma frequency is set to *ω_p_*(*σ*_0_) = 1.4 × 10^15^ rad/s. When the conductivity is set to *σ* = 2 × 10^2^ S/m, it is in the insulating state, and when the conductivity is set to *σ* = 2 × 10^5^ S/m, it is in the metallic state.

When studying the optical properties of chiral metasurfaces in the terahertz band, the Jones matrix [33,34,35] is often used in academic circles to describe the reflection characteristics of metasurfaces on incident light. This matrix correlates the incident light electric field vector with the reflected light electric field vector, and the specific expression is as follows:

(3) $$\left(\begin{array}{c} E_{r}^{L}\\ E_{r}^{R} \end{array}\right)=R_{c}\left(\begin{array}{c} E_{i}^{L}\\ E_{i}^{R} \end{array}\right)=\left(\begin{array}{cc} R_{LL} & R_{LR}\\ R_{RL} & R_{RR} \end{array}\right)\left(\begin{array}{c} E_{i}^{L}\\ E_{i}^{R} \end{array}\right)$$

In the formula, $E_{i}^{L}$ and $E_{i}^{R}$ represent the incident LCP and RCP waves, respectively; $E_{r}^{L}$ and $E_{r}^{R}$ are the corresponding reflected waves; *R_c_* is the reflection matrix; *R_LL_* and *R_RR_* are the reflection coefficients of the same polarization; *R_LR_* and *R_RL_* are the reflection coefficients of cross-polarization. The expression of the reflection matrix depends on the function and design parameters of the metasurface, which is reflected in the reflection coefficients *R_LR_* and *R_RL_*. The core performance index (CD) value of the chiral absorptive metasurface can be derived through Jones to establish the correlation between theoretical characterization and performance quantification. According to the definition of the CD effect, its essence is the absorption difference between different polarized waves, namely LCP and RCP waves. The absorptivity satisfies a square relationship with the modulus of the same-polarization reflection coefficient, and the transmission loss is ignored, as the transmitted wave is completely reflected by the bottom gold film:

(4) $$A=1-{\left|R\right|}^{2}$$

Therefore, the theoretical expression of the CD value can be derived from the Jones matrix as follows:

(5) $$CD=\left|A_{RCP}\right|-\left|A_{LCP}\right|=\left|\left(1-{\left|R_{RR}\right|}^{2})-(1-{\left|R_{LL}\right|}^{2}\right)\right|=\left|{\left|R_{LL}\right|}^{2}-{\left|R_{RR}\right|}^{2}\right|$$

This derivation shows that the CD effect of the metasurface is mainly determined by the difference in the same-polarization coefficients, while the cross-polarization coefficients reflect the interference of polarization conversion on the CD effect. Therefore, the chiral structure designed in this study ensures that the difference in the same-polarization coefficients is sufficiently large, and the cross-polarization coefficients are equal and sufficiently small by regulating the symmetry, ensuring that the CD effect mainly comes from the difference in the same-polarization absorption, thereby improving the purity of chiral selectivity.

The work of this study is to create a chiral metasurface to realize the tunable chiral absorption function through temperature control. In the simulation, CST 2019 software is used to calculate the optical properties of the structure through full-wave simulation with a frequency-domain solver. The numerical simulation adopts tetrahedral meshing, the unit cells are set with unit cell boundary conditions along the *x*-axis and *y*-axis, the incident electromagnetic wave impinges perpendicularly along the Z axis, and the z-direction is an open boundary to match the actual interaction scenario between terahertz waves and the metasurface.

## 3. Results and Discussion

### 3.1. Tunability of Chiral Absorption

**Figure 2:**
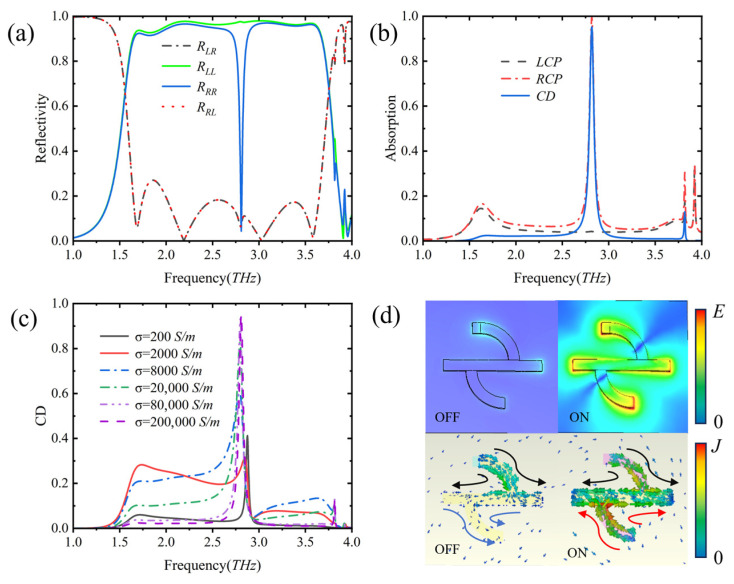
Electromagnetic response curves of the metasurface to CP polarized light, and the electric field distribution diagram of the metasurface under circularly polarized light incidence when VO_2_ is in the metallic state at the resonant frequency of 2.81 THz. (**a**) Reflection coefficients; (**b**) absorption spectrum and CD spectrum; (**c**) effect of VO_2_ conductivity on CD; (**d**) electric field and current distribution of RCP light incidence when the metasurface is in the “off” and “on” states, respectively, In the current diagram, arrows of the same color indicate identical current directions, whereas those of different colors represent opposite current directions.

The designed metal–vanadium dioxide hybrid metasurface has a “switch” characteristic for the absorption of CP light. When VO_2_ is in the metallic state, i.e., the conductivity is *σ* = 2 × 10^5^ S/m, the metasurface is in the “on” state, and the electromagnetic characteristics of the vertically incident CP wave are shown in Figure 2. It can be seen from Figure 2a that the reflection amplitude of cross-polarization remain consistent, while the reflection amplitude of the same polarization undergoes significant changes, thereby producing a strong CD effect. In Figure 2b, there is a significant absorption peak. At 2.818 THz, the absorptivity of RCP is as high as 99.4%, while the absorption of LCP is only 4.2%. Meanwhile, between 1 THz and 3.75 THz, the absorption of LCP waves is always less than 15%. This indicates that when VO_2_ is in the metallic state, the designed metasurface mainly exhibits strong reflection when facing LCP waves, while having extremely high absorptivity for incident RCP at 2.818 THz, thereby showing a significant difference in chiral selective absorption, i.e., producing a strong CD effect. Through post-processing calculation, the strong CD value reaches 0.95 at the frequency of 2.818 THz.

When VO_2_ is in the dielectric state, i.e., the conductivity is *σ* = 2 × 10^2^ S/m, the metasurface is in the “off” state, and there is no obvious CD effect at 2.818 THz. As shown in Figure 2c, it can be intuitively seen that when the VO_2_ conductivity transitions from the insulating dielectric state (conductivity *σ* = 2 × 10^2^ S/m) to the metallic state (conductivity *σ* = 2 × 10^5^ S/m), the CD curve undergoes a significant peak change in the entire frequency range, that is, the CD characteristic increases from 0.06 to 0.95 at the frequency of 2.818 THz. This change indicates that the structure has chiral tunability for circularly polarized light under different conductivities, showing its application potential in optical filtering and optical switching.

To clarify the physical mechanism of the chiral “switch”, we analyzed the electric field and current distributions of the metasurface under RCP wave incidence at the resonant frequency (2.818 THz) (Figure 2d). The core lies in the metal–insulator phase transition of vanadium dioxide (VO_2_), which directly acts as an “on–off switch” for the generation of magnetic resonance and CD. The production of strong CD relies on a magnetic dipole resonance that is selectively excited by RCP waves, and the formation condition for this resonance is a closed current loop jointly composed of the top-layer metal structure and VO_2_ patches, where the VO_2_ patches serve as a decisive “switch” in the loop.

When the metasurface is in the “OFF” state (with a VO_2_ conductivity of *σ* = 2 × 10^2^ S/m), the local electric field and current exhibit a weak coupling synergy. Based on the Drude model, VO_2_ has few free carriers and a low plasma frequency (*ω_p_*) at this time, showing a “weak response” to terahertz waves (similar to a dielectric patch). Its high-resistance characteristic breaks this low-resistance loop, making it impossible to effectively excite the magnetic dipole mode. Consequently, the metasurface shows a weak response difference to RCP. Since magnetic resonance is not excited, the incident energy cannot be effectively localized, resulting in a uniform and weak electric field distribution that fails to drive the formation of strong and continuous induced currents. The currents are only scattered in partial metal regions, and the loop is in a broken state. As a result, the metasurface exhibits a weak response difference to RCP, with a CD value of only 0.06.

When the metasurface is in the “on” state (VO_2_ conductivity *σ* = 2 × 10^5^ S/m), the local electric field–current exhibits a strong coupling synergy effect. At this time, the conductivity of VO_2_ increases by three orders of magnitude, the free carrier density and mobility are significantly enhanced, the plasma frequency (*ω_p_*) increases, showing a “strong response” to terahertz waves (similar to a metal patch). This enhances the coupling condition between the electric field and the current, making the VO_2_ patch an efficient charge transport channel, thereby breaking the geometric symmetry of the metasurface unit and strengthening the resonant coupling between the chiral structure and RCP light, that is, the local electric field exhibits a significant “strong coupling effect”. At this time, the electric field intensity is concentrated and enhanced at the junction edge of the VO_2_ patch and the quarter-circular arc, and the edge region of the metal strip. This phenomenon originates from the full excitation of the electric dipole–magnetic dipole resonance coupling of the chiral supercell—the high conductivity of metallic VO_2_ improves the matching degree between the equivalent impedance of the structure and the incident impedance of the RCP wave, and the energy of the incident electromagnetic wave is efficiently confined to the subwavelength region of the micro/nanostructure, forming a strong local field enhancement, which provides an energy basis for charge separation and transport. At the same time, this electric field distribution makes the current density show a continuous and dense distribution characteristic, and the current mainly flows along the closed path composed of the metal strip, the quarter-circular arc, and the VO_2_ patch. The low-resistance characteristic of metallic VO_2_ reduces the energy loss of carrier transport, enabling electrons to form a strong directional current under the drive of the electric field of RCP light. The Joule heating effect of this current is the core physical mechanism for the metasurface to achieve a high absorptivity of 99.4% for RCP light. At this time, the strong coupling state between current and electric field enhances the chiral symmetry breaking effect of the metasurface unit, and the CD value is 0.95. Therefore, it can be concluded that the phase state of VO_2_ regulates the degree of structural chiral symmetry breaking and carrier transport efficiency, thereby dominating the resonant coupling intensity and energy dissipation efficiency of RCP light, providing direct microscopic evidence for chiral selective regulation in the terahertz band.

**Figure 3:**
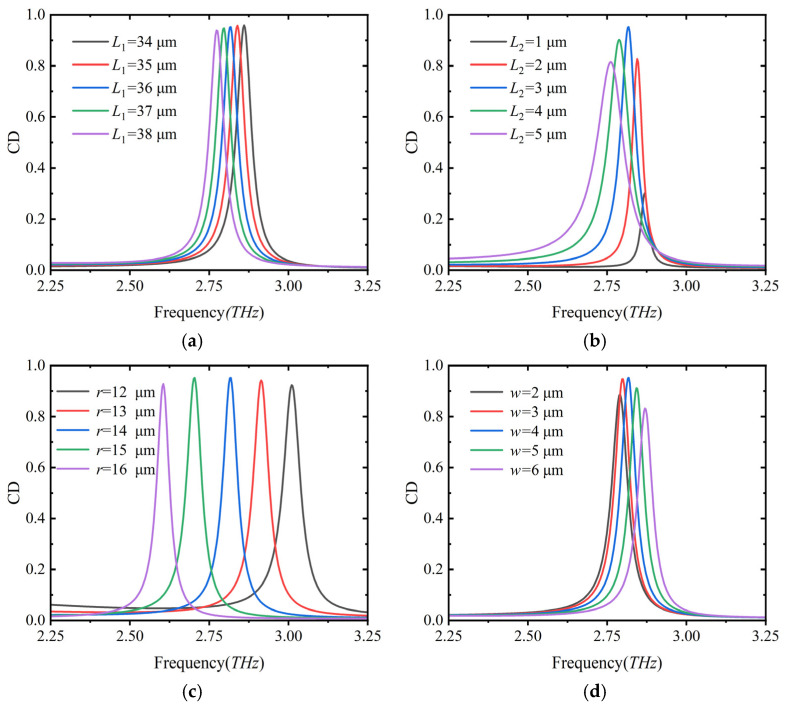
Influence of changing structural parameters on CD. (**a**) Length of the rectangular gold patch *L*_1_; (**b**) length of the rectangular VO_2_ patch *L*_2_; (**c**) radius of the metal patch *r*; (**d**) width of the metal patch *w*.

In addition, it is also important to study the influence of device parameter changes on the selective absorption of the metasurface structure. As shown in Figure 3, four main parameters affecting the CD value are displayed: the length of the rectangular gold strip *L*_1_, the length of VO_2_ *L*_2_, the radius of the quarter-circular ring *r*, and the pattern width *w*. The parameters of the control group are *L*_1_ = 35 μm, *L*_2_ = 3 μm, *r* = 14 μm, and *w* = 4 μm. When changing one parameter, the other values remain optimal.

As shown in Figure 3a, as the parameter *L*_1_ increases from 34 μm to 38 μm, the CD peak redshifts to the low-frequency direction, and the peak value first increases and then gradually decreases. When analyzing its physical mechanism, the increase of *L*_1_ improves the matching degree of the electric dipole resonance of the chiral unit with the incident RCP light. When *L*_1_ exceeds the critical value, the excessively long *L*_1_ destroys this coupling effect and prolongs the transmission energy of carriers; it can be seen from Figure 3b that as the parameter *L*_2_ increases from 1 μm to 5 μm, the CD peak continues to redshift and the width gradually broadens, and the CD peak first rises and then falls with a large amplitude span. Especially when *L*_2_ increases from 1 μm to 2 μm, the CD peak spans from 0.3 to 0.89. Here, *L*_2_ is a key factor for the metasurface structure to break the chiral structure. *L*_2_ regulates the magnetic dipole moment coupling intensity of the chiral unit—when *L*_2_ increases, the proportion of asymmetry of the unit increases, and the coupling effect between the electric dipole moment and the magnetic dipole moment is enhanced, leading to the redshift of the resonant mode frequency and mode broadening, which is ultimately reflected in the redshift and broadening of the CD peak. It can be seen from Figure 3c that as the radius *r* of the two quarter-circles increases from 12 μm to 16 μm, the CD peak redshifts to the low-frequency direction, and the spacing between the peaks is large. Through analysis, as *r* increases, the degree of chiral symmetry breaking of the unit resonates and couples with the RCP wave. After reaching the critical value, the degree of resonant coupling decreases. It can be seen from Figure 3d that as the metasurface pattern width *w* increases from 2 μm to 6 μm, the CD peak blueshifts to the high-frequency direction, and the peak value first increases and then decreases. The internal physical mechanism is that when *w* increases, the equivalent capacitance of the metal structure decreases (the increase in line width relatively reduces the electrode gap of the structure), and the chiral resonance is inversely proportional to the square root of the equivalent capacitance. Therefore, the resonant frequency increases with the increase in *w*, showing a blueshift of the CD peak. At the same time, when *w* reaches the critical value, the excessively wide metal line will increase the equivalent inductance of the structure, destroy the matching of the electric dipole–magnetic dipole resonance coupling, and trigger multimode interference, leading to a gradual decrease in the CD value. By adjusting these structural parameters, the CD effect of the metasurface in the terahertz band can be effectively optimized.

### 3.2. Wavefront Regulation

**Figure 4:**
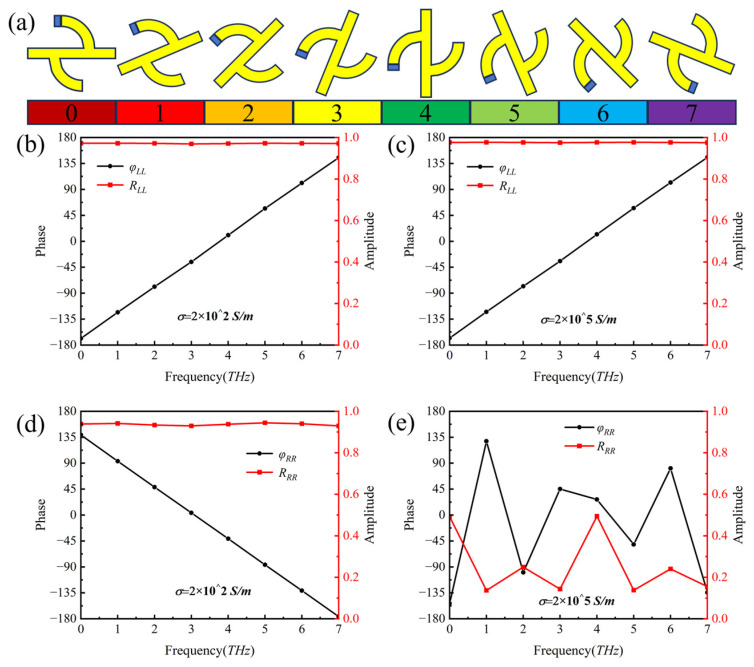
(**a**) Top-view of eight metasurface elements numbered from 0 to 7; (**b**) reflection amplitude and phase under LCP wave incidence when the conductivity of VO_2_ is *σ* = 2 × 10^2^ S/m; (**c**) reflection amplitude and phase under LCP wave incidence at *σ* = 2 × 10^5^ S/m; (**d**) reflection amplitude and phase under RCP wave incidence at *σ* = 2 × 10^2^ S/m; and (**e**) reflection amplitude and phase under RCP wave incidence at *σ* = 2 × 10^5^ S/m.

In this section, the application of the metal-VO_2_ hybrid chiral metasurface will be verified to realize the selective periodic phase modulation of CP waves in its “on” state (VO_2_ is in the metallic state). Near the frequency point of 2.80 THz, Figure 4a shows eight chiral structures with different rotation angles corresponding to the chiral metasurface covering 0° to 360° (the rotation angle between adjacent structures differs by 22.5°).

Based on the PB phase principle (when the supercell is rotated by θ, the reflected circularly polarized wave carries a phase of 2θ), the incident circularly polarized wave is encoded to achieve phase coverage from 0° to 360°. Combined with the analysis of Figure 4b,d, it can be concluded that when VO_2_ is in the insulating state, whether it is LCP wave incidence or RCP wave incidence, the reflection amplitude is stable above 0.95, and the PB phase is satisfied.

In contrast, when VO_2_ is in the metallic state, as analyzed in Figure 4c,e, for LCP waves, the reflection amplitude also remains above 0.95 and the PB phase condition is satisfied; however, for RCP waves, among the eight meta-units near the frequency of 2.80 THz, the reflection amplitudes of six meta-units are less than 0.3, and those of the other two are less than 0.5, which fails to meet the PB phase condition. Therefore, when the metasurface is in the “on” state, effective manipulation of RCP waves cannot be achieved.

**Figure 5:**
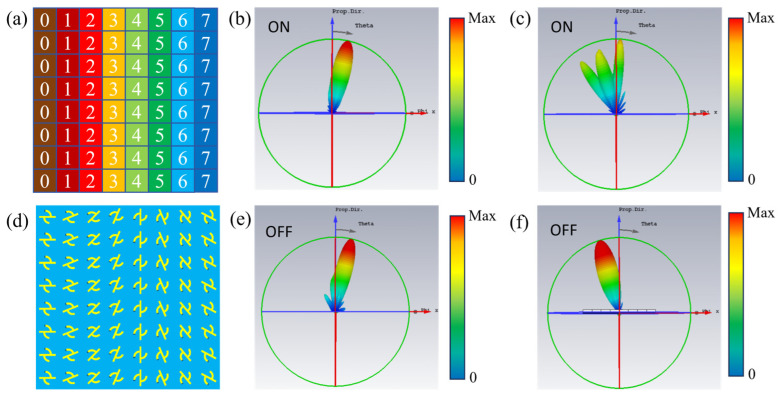
Far-field diagrams of selective absorption of beam deflection by the encoded metasurface. (**a**,**d**) Phase diagram and actual arrangement structure diagram of the beam deflection metasurface; (**b**,**e**) Far-field radiation diagram of LCP wave incidence; (**c**,**f**) Far-field radiation diagram of RCP wave incidence.

Based on the above eight supercells, the phase distribution diagram and the actual supercell arrangement structure diagram of a phase gradient metasurface are designed as shown in Figure 5a,d. The phase diagram is designed based on eight chiral supercells covering a continuous phase of 0°~360°, and a preset phase gradient is constructed through the periodic arrangement of the rotation angles of the supercells. According to Snell’s [36,37,38,39] law, the direction of the reflected wave can be changed by introducing an appropriate phase gradient on the metasurface, and its mathematical relationship is expressed as follows:

(6) $$k_{x}=-\xi -k_{0}\sin\theta_{i}$$

(7) $$\theta_{r}=\sin^{-1}(k_{x}/k_{0})$$

Among them, *θ_i_* and *θ_r_* represent the incident angle and reflection angle of the plane wave, respectively. *ξ* = 2π/(8*P*) is the phase gradient, where *P* is the period size of the supercell. *k*_0_ = 2π/*λ* represents the wave vector, and *λ* represents the wavelength of the incident wave. The theoretical deflection angle of the LCP wave is calculated to be 15°.

When LCP waves are normally incident, the metasurface exhibits stable and efficient beam manipulation characteristics regardless of whether it is in the “ON” state (vanadium dioxide (VO_2_) in the metallic phase, Figure 5b) or the “OFF” state (VO_2_ in the insulating phase, Figure 5e). In both states, the energy of the LCP waves in the far-field radiation pattern maintains a significant directional concentrated distribution without energy dispersion or beam distortion; the main lobe of the corresponding far-field radiation pattern has a clear outline, with deflection angles of 13.5° (metallic phase) and 13.6° (insulating phase), respectively, which are highly consistent with the theoretical deflection angle of 15° derived based on the PB phase principle. This deviation is mainly attributed to the diffraction effect caused by the finite array size, the non-ideal uniformity of the PB response of meta-atoms, and the electromagnetic crosstalk between units. This deviation is within an acceptable range and does not affect the core conclusion of this paper regarding chiral-selective beam manipulation. Experiments show that when VO_2_ is in the metallic and insulating phases, the reflection amplitude of the LCP waves is stably maintained above 0.95 and strictly satisfies the PB phase modulation conditions, which confirms that the beam deflection regulation of LCP waves by the metasurface has reliable stability and accurate effectiveness, and is not affected by the phase transition of VO_2_.

When RCP waves are normally incident, the far-field radiation characteristics of the metasurface show significant VO_2_ phase dependence. In the “off” state (VO_2_ is in the insulating state, Figure 5f), the RCP wave energy shows a clear directional concentrated distribution, the main lobe of the far-field directional diagram is clear, the deflection law is consistent with that when LCP waves are incident in the same state, and the reflection amplitude is stable above 0.95, meeting the PB phase modulation requirements and realizing efficient beam deflection; while in the “on” state (VO_2_ is in the metallic state, Figure 5c), the metasurface exhibits strong chiral selective absorption characteristics: the reflection amplitude of the RCP wave is significantly attenuated (six out of eight supercells have amplitudes < 0.3, two have amplitudes < 0.5) and the PB phase matching condition is not satisfied, resulting in no directional convergence effect of energy in the far-field diagram, showing obvious dispersion distribution and irregular deflection; the peak value of the electric field strength is greatly reduced, the directional diagram has no clear main lobe, and the beam deflection function fails, which intuitively confirms the chiral selective beam splitting capability of the metasurface in the metallic state.

In summary, the beam deflection regulation performance of the LCP wave is not affected by the phase transition of VO_2_, and always maintains high efficiency and stability; while the far-field radiation characteristics of the RCP wave show significant phase dependence, effective beam deflection can be achieved in the insulating state, and the deflection fails due to selective absorption in the metallic state, fully confirming the selective regulation capability of the metasurface for chiral circularly polarized waves.

## 4. Conclusions

In this paper, an MIM structured chiral metasurface based on vanadium dioxide (VO_2_) is designed. Through the synergistic effect of the metal–insulator transition (MIT) characteristic of VO_2_ and the Pancharatnam–Berry (PB) phase regulation principle, efficient dynamic regulation of chiral light in the terahertz band is realized. The research shows that by temperature-driven transition of VO_2_ conductivity between 2 × 10^2^ S/m (insulating state) and 2 × 10^5^ S/m (metallic state), the CD of the metasurface at the frequency of 2.81 THz can be continuously tuned from 0.06 to 0.95, achieving a high-contrast chiral absorption “switch” effect. Its core mechanism originates from the regulation of the phase state of VO_2_ on the degree of structural chiral breaking and carrier transport efficiency, thereby dominating the resonant coupling and energy dissipation of RCP waves.

In terms of beam regulation, the metasurface exhibits significant chiral dependence: the reflection amplitude of LCP waves is always stably maintained above 0.95, not affected by the phase state of VO_2_. Beam deflection of approximately 13.5° can be achieved through periodic phase gradient design, verifying the stability and reliability of PB phase regulation; while the RCP wave is partially selectively absorbed when VO_2_ is in the metallic state, the beam deflection function fails, and effective deflection can be achieved in the insulating state, forming a “chiral selective beam splitting” characteristic.

This design realizes the functional integration of absorptive CD tuning and reflective beam shaping with a simple MIM structure, overcoming the limitations of fixed performance and scattered functions of traditional chiral metasurfaces. It provides a compact and efficient solution for the flexible manipulation of circular polarization states in the terahertz band, and has important application potential in reconfigurable imaging, unidirectional communication, and integrated photonics systems.

## Data Availability

Data underlying the results presented in this paper are not publicly available at this time but may be obtained from the authors upon reasonable request.
